# Factors That Influence the Extensional Rheological Property of Saliva

**DOI:** 10.1371/journal.pone.0135792

**Published:** 2015-08-25

**Authors:** Amrita Vijay, Taichi Inui, Michael Dodds, Gordon Proctor, Guy Carpenter

**Affiliations:** 1 King’s College London Dental Institute, Salivary Research Unit, London, United Kingdom; 2 Wm. Wrigley Jr. Co., Chicago, Illinois, United States of America; School of Medicine and Health Sciences, University of North Dakota, UNITED STATES

## Abstract

The spinnbarkeit of saliva reflects the ability of saliva to adhere to surfaces within the mouth, thereby serving as a protective role and aiding in lubrication. Therefore, alterations in the extensional rheology of saliva may result in the loss in adhesiveness or the ability to bind onto surfaces. Mucin glycoproteins and their structures are known to be important factors for the extensional rheological properties of saliva. The conformation of mucin depends on factors such as pH and ionic strength. Chewing is one of the main stimuli for salivary secretion but creates significant sheer stress on the salivary film which could influence mouthfeel perceptions. The current study investigates the possible factors which affect the extensional rheological properties of saliva by comparing submandibular/sublingual saliva with different oral stimuli within the same group of subjects. Unstimulated and stimulated saliva (chew, smell and taste) salivas were collected primarily from submandibular/sublingual glands. The saliva samples were measured for Spinnbarkeit followed by the measuring mucin, total protein, total calcium and bicarbonate concentrations. The results indicated correlations between rheological properties and mucin/ion concentrations. However, chewing stimulated submandibular/sublingual saliva is shown to have significantly lower Spinnbarkeit, but factors such as mucin, protein and calcium concentrations did not account for this variation. Analysis of the concentration of bicarbonate and pH appears to suggest that it has a prominent effect on extensional rheology of saliva.

## Introduction

Saliva is an aqueous mixture of mucins, lipids, proteins and other bioactive molecules that coats the oral cavity [1]. Saliva has important rheological properties that may affect mouthfeel and other sensory perceptions. Saliva being a complex biological fluid possesses both surface as well as bulk rheological properties. The rheological properties of saliva are expected to provide a protective function within the oral cavity, lubricating surfaces, mouth-feel and texture perception [2].

The spinnbarkeit of saliva reflects the ability of saliva to adhere to surfaces within the mouth, thereby serving as a protective role and aiding in lubrication. Therefore, alterations in the spinnbarkeit of saliva may result in the loss in adhesiveness or the ability to bind onto surfaces which may correlate to the oral dryness, associated with Sjogren’s syndrome and oral mucositis [3]. The interfacial or surface rheological properties of saliva such as surface tension give an indication of the stability of the liquid film formed. Mucin glycoproteins are thought to be the most closely involved in the rheological properties of saliva [4], whereas statherin appears to be more important for surface properties [5]. Surface rheological properties such as interfacial tension and surface dilatational modulus of saliva can help develop an understanding of the air/water interface of the salivary film in the mouth [6]. In addition, the contact angle of saliva can reflect the degree of wetting of saliva on surfaces and hence gives an insight into the interaction of saliva with surfaces.

The spinnbarkeit of saliva is expected to arise from the presence of high molecular weight glycoproteins (mucins) that aggregate end-to-end [7,8]. Mucin glycoproteins and their structures are known to be important factors for the extensional rheological properties of saliva [9].The conformation of mucin depends on factors such as pH and ionic strength [7]. Mucins also play an important role in the wetness of the mucosal surfaces and this is shown to have an effect on maintaining the hydration of the oral mucosa [10–12]. Thus it is important to know the conformation as well as its concentration to determine its functional ability. Lubrication being an important physiological function of saliva is a function of the rheological characteristics and the surface associated components (salivary films) on one hand and the bulk components and their interplay on the other hand [13]. Therefore understanding the physical properties of saliva in relation to bulk as well surface rheology could provide important benefits and applications, however no study has been done to date, to the authors’ best knowledge, that investigated the factors influencing spinnbarkeit of saliva. It has been reported by that the extentional property (spinnbarkeit) is independent of flow rate as although there were no significant differences in flow rates between citric acid induced stimulation and mechanical stimulation, spinnbarkeit of mechanical stimulation seemed to be lower [8]. The rheology of saliva is highly dependent on the method of stimulation [14]. Different rheological properties have been identified in saliva produced by the different salivary glands, with mucin-rich submandibular/sublingual secretions being most viscous and viscoelastic, and parotid saliva secretions being the least viscous and viscoelastic saliva. These various salivary secretions contribute to rheology of WMS and contribute to its viscoelasticity and extensional rheology, aiding in the maintenance of a normal mouthfeel. In addition, submandibular/sublingual salivas, have varying concentrations of proteins when stimulated by smell, chewing or taste in comparison to unstimulated saliva. Chewing is one of the main stimuli for salivary secretion but creates significant sheer stress on the salivary film which could influence mouthfeel perceptions. The purpose of the current study was to investigate the possible factors which affect the spinnbarkeit of saliva by comparing submandibular/sublingual saliva with different oral stimuli within the same group of subjects.

## Materials and Methods

The study protocol was approved by King’s College London (KCL) College Research Ethics Committee (CREC), and all subjects enrolled in the study signed an informed consent form.

### Saliva collection

Unstimulated and stimulated salivas from the submandibular/ sublingual (SMSL) glands were collected by the drooling method from 5 volunteers who had healthy, natural dentition in 20ml universal tubes. Saliva collection was carried out in the mornings and the volunteers were advised not to eat or drink 1 hour prior to collection. Parotid saliva was absorbed using cotton rolls placed over the parotid duct orifices. Chewing (1cm rubber tube), taste (0.1M monosodium glutamate) and smell (by smelling 30%w/v freshly prepared stock solution) (Bisto Gravy Granules, Surrey, UK) stimulated salivas were collected for 5 minutes each, and flow rates were determined. Ten minute interval was maintained between each collection. Labial saliva (minor gland) was collected by direct pipetting of beads of saliva formed after first drying the lower labial surface. Saliva required for the analysis of bicarbonate was collected in a closed system using a syringe without the needle, which was placed directly into the mouth and capped soon after collection. The samples were stored on ice soon after collection and analysed individually within 5 minutes of collection.

### Semi-quantification of mucin proteins

MUC5b and MUC7a mucin glycoproteins were semi-quantified using purified mucin fractions of known concentrations (Malmo University, Sweden). The purified mucin fractions were serially diluted and were electrophoresed, and stained with Periodic Acid Schiff (PAS). Images of the PAS stained gel were analysed for band intensity using the ImageJ software. The bands were converted to peaks and the area under each curve gave the pixel intensity. Standard curves and linear equations were generated for mucins (MUC5b and MUC7a) concentration against pixel intensity as previously done [10].

### Rheology

#### Extensional rheology

The spinnbarkeit was measured using the Neva Meter (Ishikawa Iron Works, Japan).The device uses the electrical resistance of liquids produced by the application of a constant voltage reached infinity to detect the point at which the liquid thread is severed [15]. The stretching rate was set at 25% and the sample volume was 50μl. For more accurate spinnbarkeit results, the dry mode was selected and the measuring probe was wiped dry before each measurement. Measurements were taken five times, and the spinnbarkeit was determined by calculating the averages of the five values.

#### Interfacial rheology

The Interfacial tension (IFT) and dilational modulus (DM) was determined by analysing the profile of a droplet of saliva (10μl) using the Laplace equation. The measurements were made using the pendant drop analyzer (FTA1000 Drop Shape Instrument, First Ten Angstroms, Cambridge, UK) according to manufacturer’s instructions over 600s. The drop was oscillated by 5% volume throughout the experiment every 10 s, in order to obtain the DM which is an indication of the viscoelasticity of the saliva. The surface dilational modulus was determined as the ratio between the variation of surface tension and the relative change in the surface area [16].

#### Measuring the contact angle

Contact angle measurements were made by placing a droplet of saliva (10μl) onto a clean glass slide, allowing 30 s to settle and the contact angle (θ) made by the droplet or the angle at which the liquid meets the solid surface using the pendant drop analyzer (FTA1000 Drop Shape Instrument, First Ten Angstroms, Cambridge, UK).

### Statistical analysis

Statistical significance was tested using one-way ANOVA and two sample t test. Relationships yielding p-values less than 0.05 were considered significant. All values were expressed as the mean ± SE.

## Results

The mean salivary flow rates were significantly different within the SM/SL samples with unstimulated saliva showing the lowest (0.21±0.03 ml/min) and the highest flow rate for the taste stimulated samples (1.16 ± 0.5ml/min) (Table 1). The flow rate of labial saliva from the lower lip was 100 fold less than SM/SL samples (p = 0.0001). There were no significant differences in the concentration of total protein and calcium in the SM/SL saliva samples. However, the concentration of bicarbonate for the chew sample was significantly higher (p = 0.003) compared to the remaining test samples. The concentration of bicarbonate in whole saliva is dependent on flow rate [17]. However, the concentration of bicarbonate for the chew sample was significantly higher although its flow rate (1.12ml/min) was lower than that of the taste sample (2.04ml/min). This was also reflective in the pH measurements (Table 2). The bicarbonate concentration of labial saliva could not be measured because the minimum volume of saliva required could not be obtained. The concentrations of MUC5B and MUC7 remained broadly similar in all the SM/SL test samples. However, the concentration of MUC5B and MUC7 was significantly higher (p = 0.0001, p = 0.004) in labial saliva compared to the SM/SL samples.

**Table 1:** Flow rate and concentrations of total protein, calcium, bicarbonate, mucin concentration of unstimulated and stimulated SM/SL saliva and labial saliva.

Saliva (n = 5)	Flow rate (ml/min)	Protein concentration (mg/ml)	Calcium concentration (mmol/l)	Bicarbonate concentration (mmol/l)	MUC5B concentration (μg/ml)	MUC7 concentration (μg/ml)
Unstimulated SM/SL	0.21 ±0.03*	3.86±0.55	2.146±0.23	1.86±0.28	285.34±4.28	201.78±4.18
Chew SM/SL	0.68 ±0.1	2.94±0.41	1.664±0.91	5.97±0.71**	205.15±5.78	175.34±5.18
Smell SM/SL	0.51 ±0.04	3.26±0.49	1.987±0.46	2.51±0.26	212.23±5.38	130.45±4.78
Taste SM/SL	1.16 ±0.09*	2.16±0.42	1.815±0.02	3.86±0.98	218.45± 4.88	152.65±4.38
Labial	0.002±0.02***	1.78±0.27*	2.263±0.81	1.06 ± 0.53	438.67±3.88***	265.56±2.78**

Mean ±SEM. (n = 5).

*p< 0.05,

**p< 0.005,

***p<0.0001

**Table 2:** Interfacial, extensional rheology and pH measurements for unstimulated and stimulated SM/SL saliva and labial saliva.

Sample	Surface tension (N/m)	Dilatational modulus (mN/m)	Spinnbarkeit (mm)	Contact angle (θ)	pH
Unstimulated SM/SL	51±0.79	65.46±2.17	38.1±5.32*	66±1.19	6.6±1.23
Chew SM/SL	51.66±0.55	41.77±1.84*	8.6±3.41**	77.4±1.41	7.9±1.56
Smell SM/SL	52.11±0.36	58.99±2.26	27.1±4.34	70.3±1.42	6.8±1.34
Taste SM/SL	51.03±0.45	49.47±2.22	16.6±3.21	69.7±1.12	7.2±1.67
Labial	60.62±0.21 ***	68.10±1.18	61.1±3.65***	54±1.52**	6.3±1.21

Mean ±SEM. (n = 5).

*p<0.05,

**p<0.005,

***p< 0.0005

The surface tensions of all unstimulated/stimulated SM/SL saliva samples, as determined by the pendant drop analyser were very similar. However, there were no significant differences between the SM/SL samples. The dilatation modulus of saliva showed a significant decrease (p = 0.03) for the chew sample compared to resting, smell, taste and labial saliva.

The contact angle measurements of chewing stimulated SM/SL saliva were higher compared to saliva at rest and other saliva stimulated test samples indicating that chewing stimulated saliva had a lower wetting ability (Table 2). Labial saliva showed a significantly lower (greater wetting ability) contact angle compared to the rest of the test samples (p = 0.004).

The Spinnbarkeit showed a significant difference between resting and stimulated SM/SL test samples (p< 0.05). Pair wise comparison showed that chewing stimulated saliva showed a significantly lower spinnbarkeit (p<0.005) than resting saliva.

**Fig 1:**
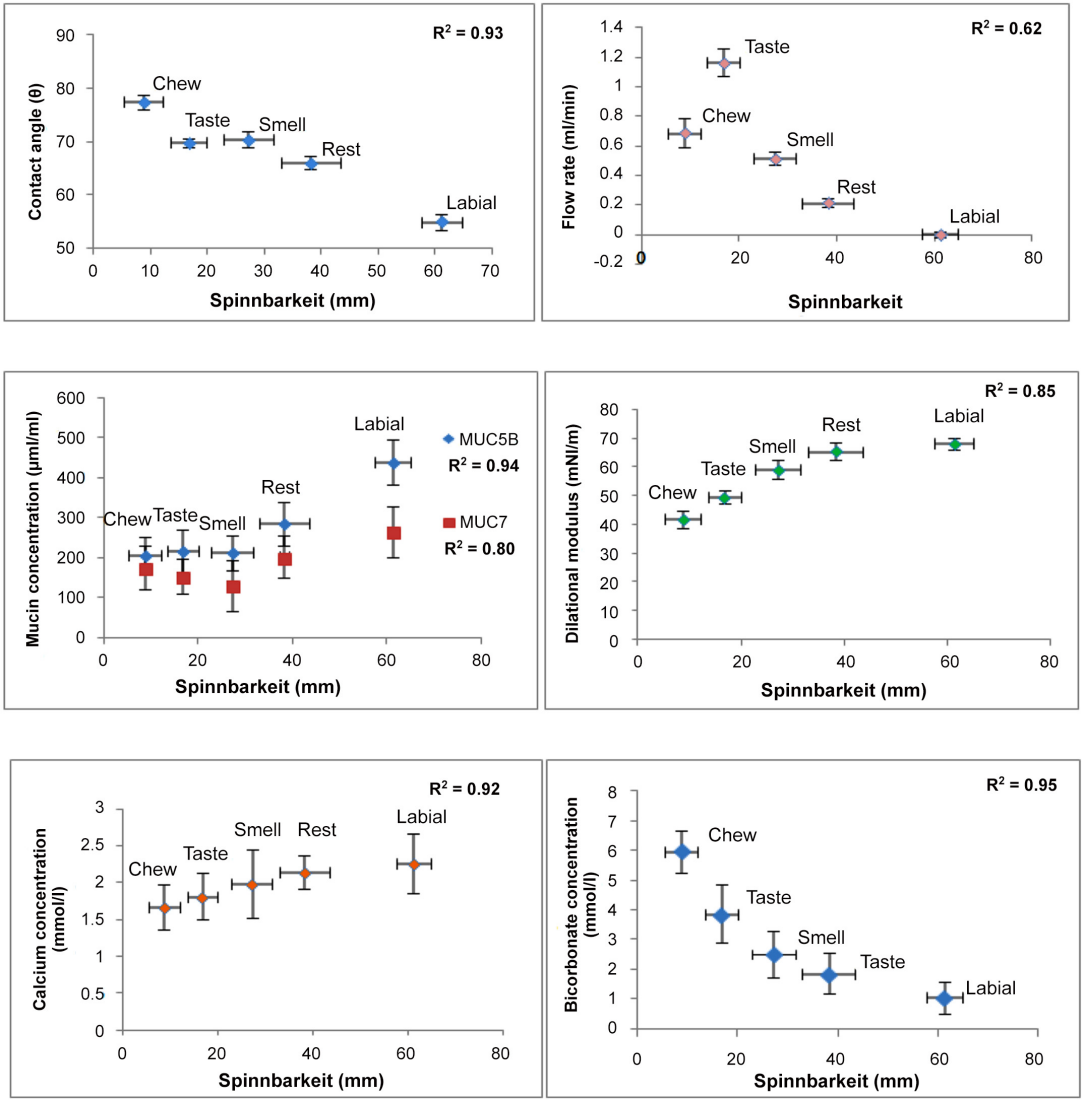
Factors showing positive and negative correlations with saliva Spinnbarkeit. (a) Correlation between contact angle and spinnbarkeit (R2 = -0.93); (b) Correlation between flow rate and spinnbarkeit (R2 = 0.62); (c) Correlation between individual mucin concentrations (MUC5B, MUC7) and spinnbarkeit (MUC5B: R2 = 0.94, MUC7: R2 = 0.80); (d) Correlation between protein concentration and spinnbarkeit (R2 = 0.07); (e) Correlation between calcium concentration and spinnbarkeit (R2 = 0.92); (f) Correlation between bicarbonate concentration and spinnbarkeit (R2 = 0.95). (Mean ± SEM. (n = 5).

Fig 1a, demonstrates the negative correlation between contact angle and spinnbarkeit for the SMSL and labial test saliva samples, where in an increase in spinnbarkeit leads to a decrease in contact angle (greater wetting ability). A similar trend was also observed for saliva flow rates as shown in Fig 1b. Fig 1c suggests that there exists a strong positive correlation between individual mucin concentrations and spinnbarkeit. However, although the concentrations of MUC5B and MUC7 are not significantly different within the stimulated SMSL saliva samples, the significant drop in spinnbarkeit of chewing stimulated saliva samples suggests that the lower elasticity is due to other factors apart from mucin content. Fig 1e and 1f show that ions such as calcium and bicarbonate concentrations are strongly correlated with Spinnbarkeit.

**Fig 2:**
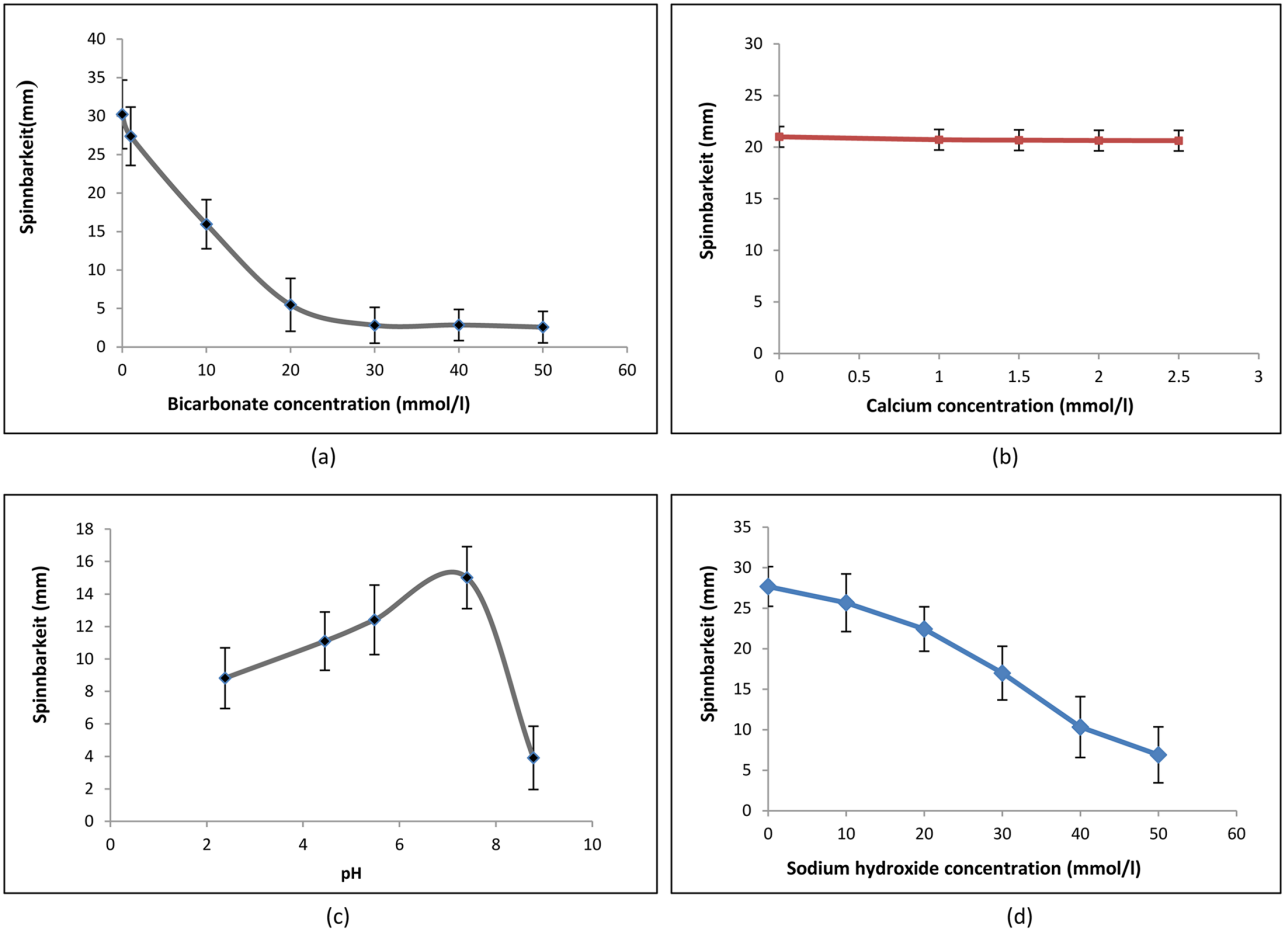
Effect of bicarbonate, calcium concentrations and pH on spinnbarkeit of unstimulated SMSL saliva sample. Mean ±SEM. Data represents results from atleast three independent experiments.

In order to further explore the influence of bicarbonate ions on the spinnbarkeit of saliva, varying concentrations of sodium bicarbonate was added directly to saliva and the spinnbarkeit was measured immediately (Fig 2a).

A rapid drop in spinnbarkeit was observed in a concentration range of 2-20mM/l added bicarbonate. The concentration of bicarbonate for the chew sample (Table 1) falls within the range required for changes in spinnbarkeit to occur. However, the spinnbarkeit of chewing stimulated saliva has much lower spinnbarkeit for the concentration of bicarbonate measured. Bicarbonate being a chelator of calicium, we tested out the addition of calcium in-vitro but showed no effect on the spinnbarkeit of unstimulated saliva (Fig 2b). Since the pH of saliva is largely governed by bicarbonate ions, the effects of pH was studied next by adding concentrated solutions of acetic acid or sodium hydroxide to freshly collected untsimulated saliva (Fig 2c). As the pH of saliva decreases, from its base at pH 7.1, the spinnbarkeit shows a gradual fall. However, the spinnbarkeit of saliva shows a steep fall when pH was increased from its baseline. This was further confirmed with the addition of sodium hydroxide in the same concentration range as sodium bicarbonate showed a similar trend in the change in spinnbarkeit (Fig 2d).

## Discussion

Extensional rheology is a property of saliva that has important implications for adhesion and lubrication [2]. The development of extensional elasticity was initially thought to arise due to the physical entanglement of mucin chains and the concentration of mucin species may therefore account for an increase in the elastic properties. A positive correlation between mucin concentration and extensional rheology was seen in the present study. However, chewing stimulated saliva was not significantly different in its mucin concentration compared to other samples but showed a significant drop in its elastic properties suggesting that mucin concentration alone cannot account for the extensional rheological properties.

Previous reports by Stokes and Davies (2007) have shown chewing stimulated saliva to have a significant drop in its elastic properties when compared to unstimulated and saliva stimulated by taste and smell. This drop in elasticity has been anticipated to be due to the stimulation of mainly the parotid gland which is a serous secretion without mucins. However, they found no significant variation of saliva viscosity with saliva secreted by different stimuli (Stokes and Davies 2007). In the present study wherein chewing stimulated saliva was collected primarily from submandibular/sublingual glands and the presence of parotid secretions was minimized. This suggests that lower elasticity of chewing stimulated saliva is due to other factors apart from reduced mucin content or contributions of other factors from parotid saliva.

One possible explanation for altered extensional rheology could be a change in the macromolecular arrangement of the mucin chains due to mechanical action of chewing or due to other factors such as the interaction of bicarbonate ions with free/bound calcium ions resulting in a change in the structural conformation of mucins. Although we cannot rule out a mechanical effect, the present study demonstrated that a drop in the spinnbarkeit of saliva as seen in the chewing sample correlated with its bicarbonate levels and pH. Although the levels of bicarbonate reported here are lower than what has been reported previously, we have used a clinically validated method of determining bicarbonate and must presume that either our collection protocol (which also compared under oil parotid collection) is flawed or that previous estimates were far too high. The persistent length and structure of mucin chains is likely to be influenced by factors such as the pH and ionic strength of the solvent [2,4].Chen et al (2010), have shown that HCO_3_^-^ directly affects mucin swelling and hydration by chelating free and mucin bound calcium ions and thereby altering its rheological properties. In other systems (lungs) bicarbonate ions behave as calcium sequesters which induce rapid molecular expansion of mucin chains [18]. Increased concentration of bicarbonate may alter the typical conformation of mucin chains to aggregate end-to-end hence altering the extensional rheological properties of saliva. Furthermore contact angle measurements were also altered which may also reflect the altered conformation of mucin chains and hence the inability of mucins to contribute to the extensional property or elastic nature which enables saliva to adhere onto surfaces.

Based on the hypothesis by Quinton and colleagues, physiologically, mucins seem to be secreted simultaneously with HCO_3_^−^ which is alkaline and forms a vital component of the pH buffering system [19]. Chen et al have also reported that the bicarbonate ion reduces the amount of free calcium and calcium bound to mucins, which enhance mucin swelling and hydration by reducing calcium cross linking in mucins, thereby decreasing its viscosity and creating a more relaxed structural arrangement. The secreted bicarbonate ion is derived from CO_2_, which is converted to HCO_3_ in the presence of a cytoplasmic carbonic anhydrase in the salivary glands. Bicarbonate ions have been known to function as the principal pH buffer system in saliva and in its regulation of saliva pH [20]. Increasing concentrations of HCO_3_^-^ would result in the pH of saliva becoming more alkaline which may cause mucins to lose the ability to crosslink resulting in a more dispersed configuration. Indeed the time-dependent loss of extensional properties in saliva may relate to the loss of bicarbonate (via CO_2_) and the alkalisation of saliva. At lower pH, mucins are known to form gel phases [21–23]. At acidic pH, (i.e. pH = 2) the carboxylates of the salt bridges are protonated, breaking the salt bridges and allowing the unfolding and exposure of hydrophobic regions which are then able to associate with adjacent molecules acting as the crosslinks of a gel [7,21]. Therefore at lower pH, hydrophobic interactions result in an increased tendency for aggregation, whereas higher pH results in the dispersion of mucin molecules [17].

The polymer network of mucus has a characteristic tangled topology [19,24–26]. The rheological properties are governed mainly by the tangle density of mucin polymers, which decreases with the degree of swelling (hydration) which critically dictates mucus rheological properties [27].

**Fig 3:**
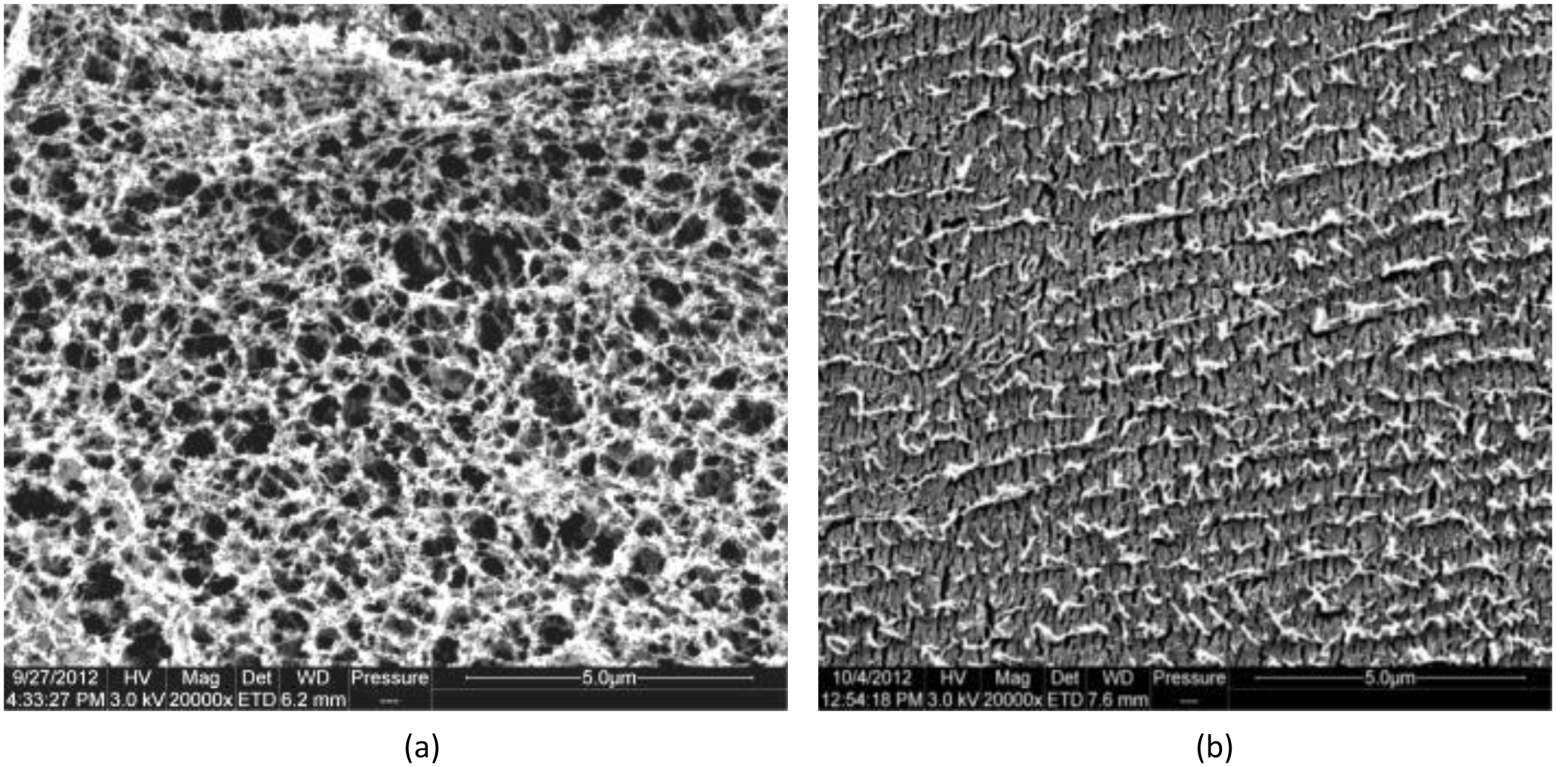
Cryo-Scanning electron micrograph images of unstimulated SMSL and parotid saliva. (a) section of unstimulated SMSL saliva showing the appearance of a filamentous mucin network; (b) section of parotid saliva showing a continuous matrix with the absence of a filamentous mucin network (Scale bar = 5μm).

The condensed network of mucin chains as shown in Fig 3, is considered to be due to crosslinking of calcium ions [27–29]. Chelating or removing these calcium ions would result in rapid swelling, hydration, and dispersion of mucin networks [30,31]. Bicarbonate ions are believed to act as a chelator of calcium ions and not only chelate free calcium ions but also mucus bound calcium ions [32]. This could explain the trend in the decrease in concentrations of total calcium shown in the present study. The complex formed between mucin subunits and non-mucin species in saliva result in the formation of an elaborate suprastructure that may be stabilised by both covalent and non covalent forces [33].

It is this suprastructure that has been demonstrated to be responsible for the unique rheological properties i.e. high elasticity, adhesiveness that are characteristics of mucin molecules [34]. The high proportion of carbohydrates in these molecules makes it likely that mucin glycoproteins presents as random coils with little secondary structure.

The conformational changes which may be caused due to the high concentration of bicarbonate ions and thereby result in massive mucin expansion by static repulsion could be studied by measuring the pore size formed within the filamentous network. A more relaxed arrangement of the filamentous network may therefore result in large pore sizes between structural aggregates. The general nature of the observed structures and pore size measurements will be evaluated further in future studies.

The high elasticity of saliva may help it to adhere to surfaces within the mouth, thereby coating the oral cavity and aiding in lubrication. The association of mucin molecules at lower pH has been reported to result in an increased adhesion of mucin molecules to oral surfaces [4]. Lubrication and salivary film coating are essential for attaining a ‘normal’ mouthfeel and plays an important role in sensory perception. Therefore, altered extensional rheology (as seen in chewing saliva) is likely to affect the functionality of saliva in terms of lubrication and surface adhesion within the oral cavity and hence impact mouthfeel. Further studies on the effect of chewing on perceived mouthfeel should be undertaken using a subjective questionnaire.

In conclusion, our results show that within the same individuals saliva’s extensional rheology can be differentiated depending on the type of sensory inputs used to stimulate the saliva. The results also show correlations between rheological properties and mucin/ion concentrations. It is evident that chewing stimulated SMSL saliva has been shown to have significantly lower spinnbarkeit, but factors such as mucin, protein and calcium concentrations did not account for this variation. Bicarbonate concentration and pH appears to suggest that it has a prominent effect on extensional rheology.
